# Mitigating lead acetate-induced histopathologic and physiologic disorders in rats receiving vitamin C and glutathione supplement

**DOI:** 10.1016/j.heliyon.2024.e41256

**Published:** 2024-12-14

**Authors:** Mohamed Gaber Shalan

**Affiliations:** Zoology and Entomology Department, Faculty of Science, Arish University, North Sinai, Egypt

**Keywords:** Lead acetate, Vitamin C, Glutathione, Apoptosis, Supplement

## Abstract

The present work examines the extreme impact of lead acetate and the preventive function of co-supplementation with vitamin C and glutathione. It hypothesizes that these supplements can alleviate the poisonous effects of lead exposure. Eighty male albino rats, weighing 100 ± 15 g, were categorized into four groups: the control group, the second group receiving daily supplements of 100 mg/kg of body weight glutathione and 1 mg/100 g of body weight vitamin C orally, the third group receiving 100 mg/kg body weight of lead acetate orally daily, and the fourth group receiving similar oral dosages of lead acetate along with glutathione and vitamin C. Lead exposure significantly decreased body weight and relative testis weight, while relative organ weights for the liver, kidney, and spleen increased significantly. Additionally, lead acetate increased plasma glutamic pyruvic transaminase and glutamic oxaloacetic transaminase activities and plasma creatinine concentration (p < 0.05). Lead concentration rose significantly in blood, urine, liver, and kidney (p < 0.05). Examinations revealed that lead acetate exposure induced apoptotic DNA fragmentation in hepatocytes, significantly increasing caspase-3 activity (91 %) and annexin V indicators. Moreover, lead exposure induced a decrease in sperm count and motility, along with an increase in abnormal sperm morphology. However, vitamin C and glutathione supplementation significantly improved these adverse impacts, suggesting their protective function in counteracting the harmful impacts of lead acetate in different organs.

## Highlights

• Lead acetate is a potentially hazardous toxin and spreads widely.
• Lead exposure triggered oxidative damage and lipid peroxidation.
• Lead acetate induces physiological and histopathological alterations in different body organs.
• Combined vitamin C and glutathione supplementation reduce lead acetate toxicity.

## 1 Introduction

Lead is widely prevalent in the environment and broadly used in various industries, particularly in producing construction materials, paints, batteries, and plumbing, contributing to increased human exposure. Lead toxicity poses a serious global health risk [1], causing severe, often irreversible organ damage; thus, investigating protective measures against lead-induced injury is critical in both clinical and environmental contexts. Elevated lead levels lead to chronic toxicity in several tissues and organs, such as the liver [[2], [3], [4]], kidneys [5,6], brain [7,8], and testes [9,10], resulting in functional and structural impairments in these vital systems. This toxicity is primarily attributed to oxidative stress, as lead increases the generation of free radicals and inhibits the activity of the antioxidant enzymes of the body [[11], [12], [13]]. Additionally, lead can replace essential cations in proteins and enzymes, causing dysfunction in their primary roles within the body [14]. Chelation-based prevention strategy for lead toxicity has limitations, including side effects and limited effectiveness in reversing organ damage [15]. Antioxidant prevention with vitamin C and glutathione offers a promising alternative: targeting oxidative stress and neutralizing free radicals-key contributors to lead toxicity.

Current research focuses on using naturally occurring, highly effective antioxidants, to alleviate the toxic effects of lead. Vitamin C is a potent antioxidant capable of neutralizing reactive oxygen species (ROS) and chelating heavy metals [16], which reduces the likelihood of lead interacting with biomolecules [17]. Vitamin C notably lowers lipid peroxidation levels, enhancing cell protection from lead-induced damage [18,19]. Research has indicated that higher serum vitamin C levels are linked to reduced blood lead concentrations, indicating its influential protective role [20].

Glutathione is a prevalent antioxidant containing a crucial sulfhydryl group, which has a significant function in neutralizing reactive oxygen and nitrogen radicals [21]. The glutathione-antioxidant system aids in converting these reactive species into non-reactive compounds, facilitated by glutathione peroxidase (GPX) and subsequently restored to reduced glutathione (GSH) by glutathione reductase (GSR) [22]. Additionally, glutathione participates in the biotransformation of toxic substances and binds to various harmful compounds through glutathione-S-transferases (GSTs), safeguarding cells from harm induced by responsive electrophiles [23,24].

Previous studies have concentrated on individual antioxidants in fighting lead acetate toxicity, but the researchers have not thoroughly investigated the combined protective impact of vitamin C and glutathione. This research examines the synergistic effects of the concurrent use of vitamin C and glutathione in alleviating lead acetate-induced toxicity, offering a more effective strategy for lead poisoning protection. Thus, this paper investigates the preventive role of the combined vitamin C and glutathione intake in mitigating lead-induced toxicity.

## 2 Materials and methods

### 2.1 Chemicals

We purchased pure lead acetate, vitamin C (L-ascorbic acid), and L-glutathione reduced (GSH) from Sigma Aldrich, Germany.

### 2.2 Preparation of the materials

1. *Lead acetate solution*: A fresh daily solution of lead acetate was constituted by dissolving 0.2 g of lead acetate in 2 ml of distilled water. Based on the animal's body weight, 100 mg/kg of body weight was given to each rat using a gastric tube (e.g., we gave the animal of 100 g body weight, 0.1 ml of lead acetate solution).
2. *Vitamin C solution*: A fresh solution of vitamin C (L-ascorbic acid) was also prepared daily by dissolving 0.02 g of pure ascorbic acid powder in 2 ml distilled water to achieve a dosage of 1 mg/100 g of body weight for each animal (e.g., we gave the animal of 100 g body weight, 0.1 ml of vitamin C solution). We carefully calculated the doses based on the animal's body weight and administered them orally using a gastric tube to prevent the oxidation and degradation of vitamin C.
3. *Glutathione solution*: A fresh L-glutathione reduction (GSH) solution was formulated each day by dissolving 0.2 g of powdered glutathione in 2 ml distilled water to reach a 100 mg/kg body weight dose for each animal. Like the other solutions, we administered the glutathione doses orally using a gastric tube to ensure accurate dosing.

### 2.3 Animals

The study involved eighty male albino rats *(Rattus norvegicus*), aged 4–5 weeks, obtained from the animal facility, Suez Canal University, weighing 100 ± 15 g, were utilized as experimental animals. We housed the animals in four groups within plastic crates. The animals were maintained under a 12-h light/dark cycle with a stable temperature of (25±2 °C) and relative humidity of (45 ± 5 %). They were provided with a conventional standard diet, and given free access to water. The animals were acclimatized to the lab conditions for ten days. The experimental protocol was reviewed and approved by the Research Ethics Committee of the Faculty of Science at Arish University with approval number “ARU/SF.02". Our experimental procedures comply with the guidelines outlined in the 8th edition of the guide for the care and use of laboratory animals with strict adherence to ethical guidelines. The study complies with all regulations.

### 2.4 Experimental design

The researcher randomly separated the animals into four groups containing twenty rats. The first group was designated as the control, receiving distilled water orally which served as the vehicle. The second group got orally 1 mg of vitamin C per 100 g of body weight [25] and 100 mg/kg body weight glutathione daily (vit C + GSH group) [26]. The third group administered 100 mg of lead acetate per kg of body weight daily, which equals 1/6 LD50 (Pb group) [27]. The fourth group got orally 1 mg/100 g vitamin C, 100 mg/kg glutathione, and 100 mg/kg lead acetate daily (vit C + GSH + Pb group).

### 2.5 Duration time

Animals were weighted using a digital balance, then killed by pithing, followed by rapid dissection and subsequent removal of tissues. Compared to the control group, we conducted comparative analyses after 1, 2, 4, and 6 weeks of treatment. These time intervals were earlier used by Guillemot-Legris et al. [28] to study inflammatory responses to high-fat diet feeding.

### 2.6 Collection of plasma samples

We drew blood from the abdominal vein into tubes containing Ethylenediaminetetraceetic acid to obtain plasma samples. Subsequently, centrifugation of the samples was carried out at 1000 g for 15 min and preserved at −30 °C for subsequent biochemical analyses.

### 2.7 Tissue preparation for microscopic and gel analysis

Following animal pithing, the liver, brain, kidney, testis, and spleen were excised, blotted dry on filter paper, and weighed using a digital balance. We divided each organ: the first portion was preserved in a 10 % formal saline solution for histological analysis; the second was instantly used for gel analysis; and the residual part was kept at −30 °C for preservation.

### 2.8 Flow cytometry

Apoptosis in liver cells was assessed using the annexin V-FITC/PI apoptosis detection kit following the producer's guidelines. Cells analysis was performed using flow cytometry (BD Bioscience, USA). The apoptosis % was calculated based on the distribution of cells in the following quadrants: viable cell LL (-ve for both stains, Q1), early apoptosis LR (annexin V^+^/PI^−^, Q2), Late apoptosis UL (annexin V^+^/PI^+^, Q3), and necrosis UR (annexin V-/PI+, Q4).

We used the FITC active caspase-3 apoptosis kit to measure caspase-3 activity in liver cells as stated by the supplier's instructions. Cells were examined using flow cytometry (BD Bioscience, USA), and the percentage of caspase-3 activity was calculated.

### 2.9 Histopathological techniques

Histological sections, each five microns thick were carefully prepared and stained with hematoxylin and eosin. Microscopic analysis was conducted for the specimen [29].

### 2.10 DNA extraction

The tissue homogenates (200 mg) underwent processing using the Zymoresearch Quick-g DNATM MiniPrep kit (Catalog No. D3024, USA) for DNA isolation. After centrifugation of the tissue homogenates at 12,000 g for 10 min at 4 °C, the resulting supernatants were utilized for DNA extraction [30].

### 2.11 Application of agarose gel electrophoresis for detecting extracted DNA

Isolated DNA was identified by agarose gel electrophoresis [31].

### 2.12 Biochemical measurements

Parameters associated with redox imbalance were analyzed using Biodiagnostic kits, Dokki, Egypt. Activity of superoxide dismutase (SOD) was determined as referenced by Nishikimi et al. [32]. Catalase (CAT) activity was determined by the method of Aebi [33]. Malondialdehyde (MDA) content in plasma was quantified utilizing the method described by Ohkawah et al. [34]. Nitric oxide concentration was quantified as described previously [35].

Activities of Glutamic pyruvic transaminase (GPT) and Glutamic oxaloacetic transaminase (GOT) were performed applying Schumann and Klauke's modified method [36].

Creatinine levels were calculated via Diamond Diagnostics kits, Holliston, USA, following the method of Heinegaard and Tiderstrom's procedure [37]. Lead concentration was determined in whole blood, urine, liver, and kidney homogenates employing a T80 UV/visible double-beam spectrophotometer [38].

### 2.13 Testicular indices

We collected sperm samples immediately following the removal of the testis. Sperm count and sperm motility were assessed using the method of Dominic and Padmaja's method [39]. Abnormal sperm morphology was evaluated using the technique of Ekaluo et al. [40].

### 2.14 Statistical analysis

The results are expressed as the means ± standard deviation (SD) for five rats in each group. A one-way analysis of variance (ANOVA) was used to examine data from the experimental and control groups. Tukey's test was used to perform multiple comparisons. The difference was deemed significant at p < 0.05. Statistical analysis was conducted using SPSS software for Windows, version 22.0.

## 3 Results

### 3.1 Weights

**Fig. 1:**
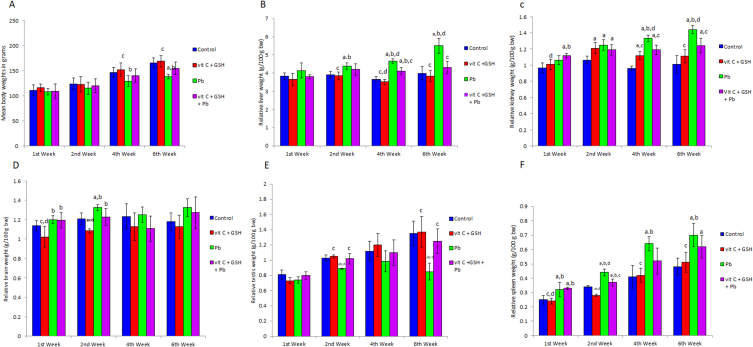
Effects of lead acetate exposure on body weight (A), relative liver weight (g/100 g bw) (B), relative kidney weight (g/100 g bw) (C), relative brain weight (g/100 g bw) (D), relative testis weight (g/100 g bw) (E) and relative spleen weight (g/100 g bw) (F) of rats. VIT C: vitamin C; GSH: glutathione; Pb: lead acetate; bw: body weight. Data are presented as means ± SD for five rats in each group. a: Significantly different than control group at p < 0.05 (Tukey's post hoc test), b: Significantly different than vitamin C and glutathione group at p < 0.05 (Tukey's post hoc test), c: Significantly different than lead acetate group at p < 0.05 (Tukey's post hoc test), d: Significantly different than lead acetate + vitamin C + glutathione group at p < 0.05 (Tukey's post hoc test).

Lead acetate evoked a significant (P < 0.05) reduction in body weight by 6 weeks of treatment (Fig. 1A). Results indicated that lead acetate induced significant elevations in the relative liver, kidney, brain, and spleen weights; however, it promoted a significant (P < 0.05) decrease in testicular weight (Fig. 1B–F). Vitamin C and glutathione supplementation improves these effects (Fig. 1A–F).

### 3.2 Biochemistry

**Fig. 2:**
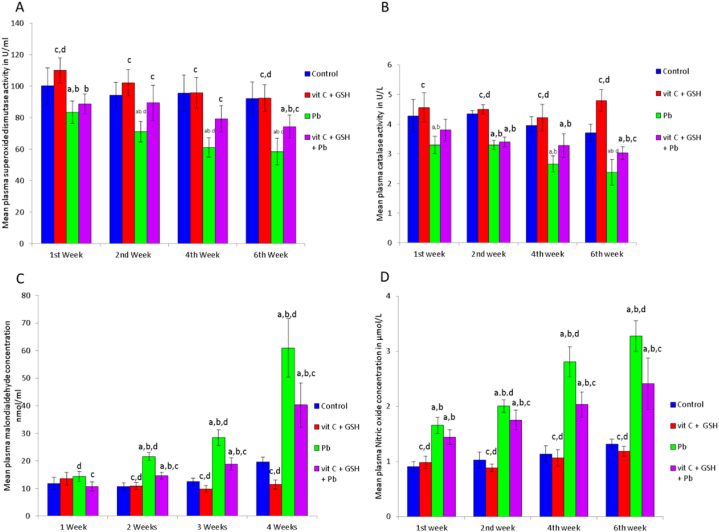
Effects of lead acetate exposure on plasma superoxide dismutase (A), catalase (B) activities, malondialdehyde (C), and nitric oxide (D) concentrations of rats. VIT C: vitamin C; GSH: glutathione; Pb: lead acetate. Data are presented as means ± SD for five rats in each group. a: Significantly different than control group at p < 0.05 (Tukey's post hoc test), b: Significantly different than vitamin C and glutathione group at p < 0.05 (Tukey's post hoc test), c: Significantly different than lead acetate group at p < 0.05 (Tukey's post hoc test), d: Significantly different than lead acetate + vitamin C + glutathione group at p < 0.05 (Tukey's post hoc test).

Chronic lead acetate toxicity caused a significant decrease in the activities of superoxide dismutase (SOD) and catalase (CAT) after 1, 2, 4, and 6 weeks of treatment, amounting to 16.7, 24.5, 36.1, and 36.5 % of normal controls, respectively, for SOD (Fig. 2A) and to 22.9, 24, 32.8, and 35.7 % of normal controls, respectively, for CAT (Fig. 2B). The results clarified that plasma malondialdehyde levels elevated significantly (p < 0.05) by 101.1, 126.8, and 210.6 % compared to normal controls in response to lead acetate exposure over 2, 4, and 6 weeks following treatment (Fig. 2C). Meanwhile, plasma nitric oxide concentrations were found to elevate significantly (p < 0.05) by 82.4, 95.1, 146.5, and 148.5 % compared with normal control rats after 1, 2, 4, and 6 weeks of treatment, respectively (Fig. 2D).

**Fig. 3:**
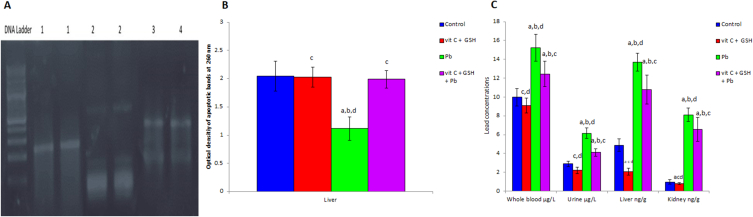
Effects of lead acetate exposure on apoptotic DNA fragmentation in rat liver after 6 weeks of treatment (A), “from left to right” (M) 1 Kp ladder; Lane 1 vitamin C + glutathione + lead acetate group; Lane 2 lead acetate group; Lane 3 vitamin C + glutathione group; Lane 4 control group, Optical density of apoptotic bands (B) at 260 nm in liver of rats after 6 weeks of lead acetate exposure, and lead acetate (C) concentrations. Data are presented as means ± SD for five rats in each group. a: Significantly different than control group at p < 0.05 (Tukey's post hoc test), b: Significantly different than vitamin C and glutathione group at p < 0.05 (Tukey's post hoc test), c: Significantly different than lead acetate group at p < 0.05 (Tukey's post hoc test), d: Significantly different than lead acetate + vitamin C + glutathione group at p < 0.05 (Tukey's post hoc test).

Lead levels increased significantly (p < 0.05) in whole blood, urine, liver, and kidney homogenates compared with normal controls after six weeks of lead acetate exposure (Fig. 3C). It could be noticed that plasma levels of GPT, GOT, and creatinine were significantly elevated (p < 0.05), especially following prolonged lead acetate toxicity (Table 1).

**Table 1:** Effects of lead acetate on GOT, GPT activities and creatinine concentration and the protective effect of vitamin C and glutathione supplementation in male albino rats.

	GPT (U/L)	GOT (U/L)	Creatinine (mg/dl)
1st week			
Control	38.6 ± 5.86	110.4 ± 11.72	0.65 ± 0.060
VIT C + GSH	35.4 ± 3.58	101.8 ± 9.60^c^^,^^d^	0.65 ± 0.100^c^
Pb	40.0 ± 2.92	137.2 ± 14.41^a^^,^^b^	0.90 ± 0.110^a^^,^^b^
VIT C + GSH + Pb	39.6 ± 5.55	125.4 ± 13.76^b^	0.72 ± 0.110
2nd week			
Control	34.4 ± 4.62	117.2 ± 11.65	0.67 ± 0.110
VIT C + GSH	32.8 ± 3.35^c^^,^^d^	112.2 ± 13.48^c^^,^^d^	0.66 ± 0.060^c^^,^^d^
Pb	55.2 ± 5.63^a^^,^^b^^,^^d^	150.2 ± 4.87^a^^,^^b^	0.98 ± 0.160^a,b^
VIT C + GSH + Pb	46.0 ± 4.47^a^^,^^b^^,^^c^	148.0 ± 16.81^a^^,^^b^	0.90 ± 0.110^a,b^
4th week			
Control	44.2 ± 6.53	121.2 ± 17.88	0.70 ± 0.110
VIT C + GSH	34.8 ± 4.60^c^^,^^d^	103.0 ± 17.36^c^^,^^d^	0.73 ± 0.070^c^
Pb	57.8 ± 7.60^a^^,^^b^	216.0 ± 18.96^a,^^b^	1.05 ± 0.120^a,b,^^d^
VIT C + GSH + Pb	56.0 ± 4.30^a^^,^^b^	183.0 ± 20.82^a,b^	0.83 ± 0.090^c^
6th week			
Control	47.4 ± 4.04	123.6 ± 14.15	0.70 ± 0.110
VIT C + GSH	39.6 ± 5.55^c^^,^^d^	125.8 ± 14.15^c^^,^^d^	0.66 ± 0.080^c,d^
Pb	68.2 ± 8.56^a^^,^^b^	244.0 ± 36.12^a^^,^^b^^,^^d^	1.23 ± 0.160^a,b,d^
VIT C + GSH + Pb	61.0 ± 6.48^a^^,^^b^	199.0 ± 14.52^a^^,^^b^^,^^c^	0.98 ± 0.090^a,b,c^

Data are presented as mean ± SD (n = 5).

a Significantly different than normal control group at *p < 0.05* (Tukey's post hoc test).

b Significantly different than vitamin C and glutathione group at *p < 0.05* (Tukey's post hoc test).

c Significantly different than lead acetate group at *p < 0.05* (Tukey's post hoc test).

d Significantly different than lead acetate + vitamin C + glutathione group at *p < 0.05* (Tukey's post hoc test).

Lead exposure induced a significant (p < 0.05) decrease in sperm count and sperm motility, particularly after six weeks of experimentation (Table 2). Conversely, the percent of abnormal sperm morphology increased significantly (p < 0.05) by 40.9, 135, 311.7, and 362.5 % after 1, 2, 4, and 6 weeks of lead acetate treatment, respectively. Administration of vitamin C and glutathione improved the biochemical indices under investigation.

**Table 2:** Effects of lead acetate on sperm parameters and the protective effect of vitamin C and glutathione supplementation in male albino rats.

	Sperm count x 10^6^/ml	Sperm motility (%)	Abnormal sperm morphology (%)
1st week			
Control	104.9 ± 15.12	87.6 ± 7.98	4.80 ± 0.83
VIT C + GSH	99.36 ± 14.74	89.0 ± 6.74	4.20 ± 0.83^c^
Pb	95.58 ± 11.51	86.2 ± 6.76	6.40 ± 1.14 ^b,d^
VIT C + GSH + Pb	100.1 ± 9.43	87.8 ± 6.30	4.40 ± 1.14^c^
2nd week			
Control	121.4 ± 10.12	91.2 ± 6.26	4.00 ± 0.70
VIT C + GSH	116.5 ± 10.11^c^	92.6 ± 5.31	4.40 ± 0.54^c^
Pb	79.56 ± 07.55^a,b,d^	83.6 ± 3.78	9.40 ± 1.14^a,b,d^
VIT C + GSH + Pb	112.0 ± 13.20 ^b^	89.2 ± 5.26	5.60 ± 0.54^a,c^
4th week			
Control	127.8 ± 13.53	92.6 ± 7.63	3.40 ± 0.89
VIT C + GSH	151.7 ± 12.76^a,c,d^	95.0 ± 6.04	3.00 ± 0.70^c,d^
Pb	83.02 ± 08.85^a,b,d^	87.4 ± 7.63	14.0 ± 1.58^a,b,d^
VIT C + GSH + Pb	106.0 ± 13.13 ^b,c^	88.2 ± 7.32	09.0 ± 1.00^a,b,c^
6th week			
Control	139.9 ± 20.77	94.4 ± 5.12	3.20 ± 0.83
VIT C + GSH	159.6 ± 17.49^c,d^	94.6 ± 7.12^c^	3.00 ± 1.00^c,d^
Pb	76.40 ± 09.16^a,b^	80.0 ± 7.90^a,b^	14.8 ± 3.95^a,b,d^
VIT C + GSH + Pb	99.36 ± 10.85^a,b^	85.8 ± 7.35	09.0 ± 2.09^a,b,c^

Data are presented as mean ± SD (n = 5). a: Significantly different than normal control group at *p < 0.05* (Tukey's post hoc test), b: Significantly different than vitamin C and glutathione group at *p < 0.05* (Tukey's post hoc test), c: Significantly different than lead acetate group at *p < 0.05* (Tukey's post hoc test), d: Significantly different than lead acetate + Vitamin C + glutathione group at *p < 0.05* (Tukey's post hoc test).

### 3.3 Molecular biology

Chronic lead consumption for 6 weeks caused DNA fragmentation in rats' livers, as reported in Fig. 3A and B. However, supplementing with vitamin C and glutathione reduced lead toxicity and was influential in repairing DNA.

### 3.4 Flow cytometry

**Fig. 4:**
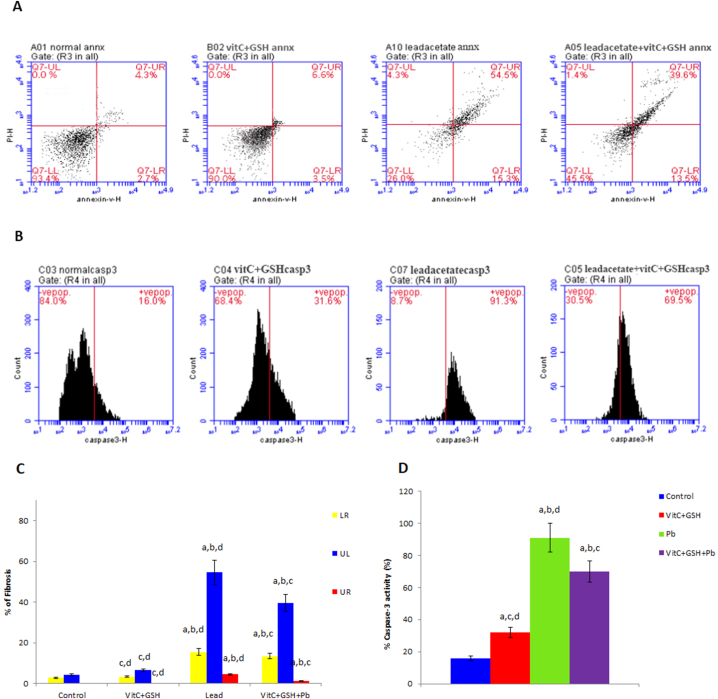
Hepatic Cell apoptosis and caspase-3 activity after 6 weeks of treatment. (A) Flow cytometry results were obtained using the annexin V/propidum iodide double staining method to detect cell apoptosis in rats' liver cells. (B) Caspase-3 activity in rats' liver cells using flow cytometry analysis. (C) Quantitative results of annexin V showing the percentage of fibrosis (LR; early fibrosis, UR; late fibrosis, UR; necrosis) in rats' liver cells in different groups under investigation. (D) Quantitative results of caspase-3 activity using flow cytometry. Data are presented as means ± SD for five rats in each group. a: Significantly different than control group at p < 0.05 (Tukey's post hoc test), b: Significantly different than vitamin C and glutathione group at p < 0.05 (Tukey's post hoc test), c: Significantly different than lead acetate group at p < 0.05 (Tukey's post hoc test), d: Significantly different than lead acetate + vitamin C + glutathione group at p < 0.05 (Tukey's post hoc test).

Apoptosis was verified via fluorescence analysis of annexin V-FITC/PI staining assay using flow cytometry. Lead acetate induced a marked increase in early and late apoptosis and necrosis of liver cells. Vitamin C and glutathione supplementation improves these effects (Fig. 4A and C). Caspase-3 activity is an essential marker of apoptotic cell death. Results in Fig. 4B and D showed that lead acetate increased caspase-3 activity percent significantly in rat's hepatocytes, confirming its role in inducing liver cell apoptosis. Supplementation of rats with vitamin C and glutathione reduces this impact.

### 3.5 Histopathological findings

**Fig. 5:**
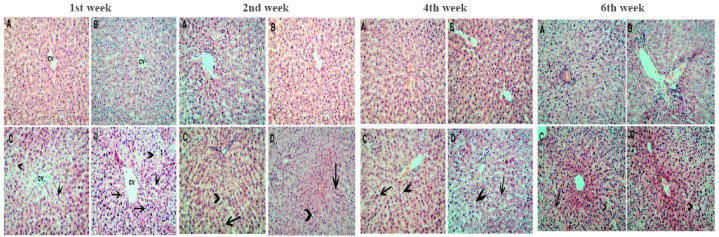
Microscopic pictures of Hematoxilin and eosin-stained liver sections. ***After 1 week***; **(A)** Control liver section showing the normal arrangement of the hepatocytes around the central vein (cv). **(B)** VIT C + GSH treated rat liver showing picture more or less similar to the controls. **(C)** Lead-exposed rat liver with signs of severe hepatocyte stress in the form of cytoplasmic vacuolation (arrowheads). **(D)** VIT C + GSH + Pb treated rat liver with signs of severe hepatocyte stress in the form of cytoplasmic vacuolation (arrowheads) and nuclear pyknosis (arrows). (Hematoxylin and eosin X 200). ***After 2 weeks***; **(A)** Control liver section showing the normal arrangement of the hepatocytes around the central vein. **(B)** VIT C + GSH treated rat liver showing picture more or less similar to the controls. **(C)** Lead-exposed rat liver with signs of severe hepatocyte stress in the form of cytoplasmic vacuolation (arrowheads) and nuclear pyknosis (arrows). **(D)** VIT C + GSH + Pb treated rat liver with signs of severe hepatocyte stress in the form of cytoplasmic vacuolation (arrowheads) and nuclear pyknosis (arrows). (Hematoxylin and eosin X 200). ***After 4 weeks***; **(A)** Control liver section showing the normal arrangement of the hepatocytes around the central vein. **(B)** VIT C + GSH treated rat liver showing picture more or less similar to the controls. **(C)** Lead-exposed rat liver with signs of severe hepatocyte stress in the form of cytoplasmic vacuolation (arrowheads) and nuclear pyknosis (arrows). **(D)** VIT C + GSH + Pb treated rat liver with signs of severe hepatocyte stress in the form of cytoplasmic vacuolation (arrowheads) and nuclear pyknosis (arrows). (Hematoxylin and eosin X 200). ***After 6 weeks***; **(A)** Control liver section showing the normal arrangement of the hepatocytes around the central vein. **(B)** VIT C + GSH treated rat liver showing picture more or less similar to the controls. **(C)** Lead-exposed rat liver with signs of hepatocyte stress in the form of cytoplasmic vacuolation (arrowheads) and nuclear pyknosis (arrows). **(D)** VIT C + GSH + Pb treated rat liver with signs of hepatocyte stress in the form of cytoplasmic vacuolation (arrowheads). (Hematoxylin and eosin X 200).

The control and vitamin C + glutathione-supplemented groups exhibited comparable histological architecture of liver cells. After one week, lead acetate-induced cytoplasmic vacuolation in hepatocytes. Then, after 2 weeks, lead promoted nuclear pyknosis. These lesions were aggravated after 3 and 4 weeks of lead toxicity (Fig. 5). These effects were mitigated by giving glutathione and vitamin C.

**Fig. 6:**
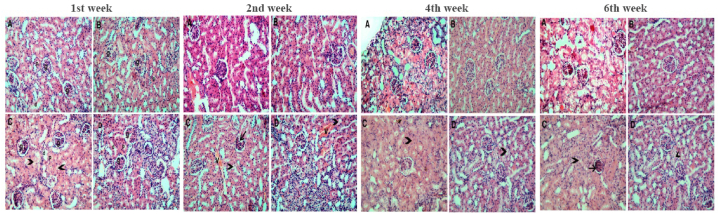
Microscopic pictures of Hematoxilin and eosin-stained kidney sections. **After *1 week****;***(A)** Control kidney section showing the normal arrangement of the kidney with the glomerulus (g), proximal convoluted tubules (p), and distal convoluted tubules (d). **(B)** VIT C + GSH treated rat kidney showing picture more or less similar to the controls. **(C)** Lead-exposed rat kidneys with almost normal glomeruli (g). The proximal convoluted tubules (p) show vacuolated cytoplasm and obliterated lumens (arrowheads). Most of the distal convoluted tubules (d) have the same picture as the proximal ones. **(D)** VIT C + GSH + Pb treated rat kidneys with almost normal glomeruli (g). The proximal convoluted tubules (p) and the distal convoluted tubules (d) are more or less closer to the controls (Hematoxylin and eosin X 200). **After *2 weeks****;***(A)** Control kidney section showing the normal arrangement of the kidney. **(B)** VIT C + GSH treated rat kidney showing picture more or less similar to the controls. **(C)** Lead-exposed treated rat kidneys with some atrophied glomeruli (arrow) with congested vasculature (V). The proximal convoluted tubules show vacuolated cytoplasm and obliterated lumens (arrowhead). Most of the distal convoluted tubules (d) have the same picture as the proximal ones. **(D)** VIT C + GSH + Pb treated rat kidneys with almost normal glomeruli with congested blood vessels (V). The proximal convoluted tubules have vacuolated cytoplasm (arrowhead). The distal convoluted tubules are more or less closer to the controls (Hematoxylin and eosin X 200). **After *4 week****s;***(A)** Control kidney section showing the normal arrangement of the kidney. **(B)** VIT C + GSH treated rat kidney showing picture more or less similar to the controls. **(C)** Lead-exposed rat kidneys with normal glomeruli. The proximal convoluted tubules show vacuolated cytoplasm and obliterated lumens (arrowhead). Most of the distal convoluted tubules (d) have the same picture as the proximal ones. **(D)** VIT C + GSH + Pb treated rat kidneys with almost normal glomeruli. The proximal convoluted tubules have vacuolated cytoplasm (arrowhead). The distal convoluted tubules are more or less closer to the controls (Hematoxylin and eosin X 200). **After *6 week****s;***(A)** Control kidney section showing the normal arrangement of the kidney. **(B)** VIT C + GSH treated rat kidney showing picture more or less similar to the controls. **(C)** Lead-exposed rat kidneys with atrophied glomerulus (arrow). The proximal convoluted tubules show vacuolated cytoplasm and obliterated lumens (arrowhead). Most of the distal convoluted tubules (d) have the same picture as the proximal ones. **(D)** VIT C + GSH + Pb treated rat kidneys with almost normal glomeruli. The proximal convoluted tubules have vacuolated cytoplasm (arrowhead). The distal convoluted tubules are more or less closer to the controls (Hematoxylin and eosin X 200).

The control and vitamin C-supplemented groups' kidneys had a typical glomerular and tubular architecture and a normal histological appearance. Following a week of exposure, lead acetate-induced cytoplasmic vacuolation destroyed the lumens of the proximal convoluted and distal renal tubules. After 2 weeks, glomeruli atrophy and vascular congestion additionally appeared. These lesions have been exacerbated later with some adaptation in the glomeruli during the 4th week of experimentation. Conversely, lead acetate-exposed rats treated with vitamin C and glutathione displayed marked improvements in these effects (Fig. 6).

**Fig. 7:**
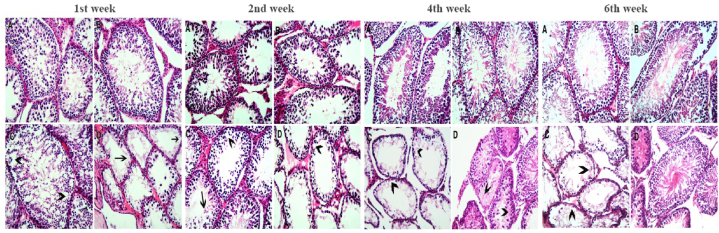
Microscopic pictures of Hematoxylin and Eosin stained testicular sections. After 1 week; (A) Control testis section showing the normal arrangement of the seminiferous tubules with the lining of spermatogonia, spermatids, and spermatozoa. The interstitial tissue appears to contain Leydig cells (L). (B) VIT C + GSH treated rat testis showing picture more or less similar to the controls. (C) Lead-exposed rat testis with signs of germinal epithelium affection in the form of intraepithelial vacuolation (arrowheads). The Leydig (L) cells appear more or less normal. (D) VIT C + GSH + Pb treated rat liver with signs of severe seminiferous tubule affection in the form of shed epithelium (arrows) and increased intertubular fibrosis. (Hematoxylin and eosin X 200). After 2 weeks; (A) Control testis section showing the normal arrangement of the seminiferous tubules with the lining of spermatogonia, spermatids, and spermatozoa. The interstitial tissue appears to contain Leydig cells. (B) VIT C + GSH treated rat testis showing picture more or less similar to the controls. (C) Lead-exposed rat testis with signs of germinal epithelium affection in the form of intraepithelial vacuolation (arrowheads). The Leydig cells appear more or less normal. (D) VIT C + GSH + Pb treated rat testis with signs of severe seminiferous tubule affection in the form of intraepithelial vacuolation (arrowheads) and increased intertubular fibrosis. (Hematoxylin and eosin X 200). After 4 weeks; (A) Control testis section showing the normal arrangement of the seminiferous tubules with the lining of spermatogonia, spermatids, and spermatozoa. The interstitial tissue appears to contain Leydig cells. (B) VIT C + GSH treated rat testis showing picture more or less similar to the controls. (C) Lead-exposed rat testis with signs of germinal epithelium affection in the form of intraepithelial vacuolation (arrowheads). The Leydig cells appear more or less normal. (D) VIT C + GSH + Pb treated rat testis with signs of severe seminiferous tubule affection in the form of intraepithelial vacuolation (arrowheads) (Hematoxylin and eosin X 200). After 6 weeks; (A) Control testis section showing the normal arrangement of the seminiferous tubules with the lining of spermatogonia, spermatids, and spermatozoa. The interstitial tissue appears to contain Leydig cells. (B) VIT C + GSH treated rat testis showing picture more or less similar to the controls. (C) Lead-exposed rat testis with signs of germinal epithelium affection in the form of intraepithelial vacuolation (arrowheads). The Leydig cells appear more or less normal. (D) VIT C + GSH + Pb treated rat liver more or less close to the controls (Hematoxylin and eosin X 200).

Histology of the control and vitamin C + glutathione group's testis reveals normal seminiferous tubules filled with spermatids and spermatozoa. Alongside normal Sertoli and Leydig cells, there are several layers of spermatocytes; the epididymal smear also shows normal sperm morphology. Lead acetate induced intraepithelial vacuolation in the germinal epithelium. The testis of rats administered vitamin C and glutathione and poisoned with lead acetate showed seminiferous tubule epithelial shedding accompanied by intertubular fibrosis in the 1st week of treatment (Fig. 7). This effect was reduced gradually with time until the testicular tissue restored standard architecture after 6 weeks of treatment.

**Fig. 8:**
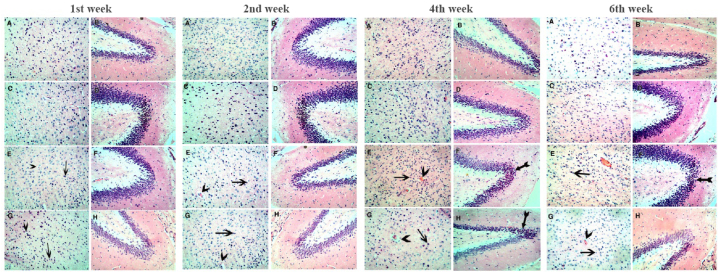
Microscopic pictures of Hematoxilin and Eosin stained brain sections. After 1 week; (A) Control rat brain section shows the normal nerve cells with dispersed chromatin and basophilic cytoplasm. (B) Control rat brain section showing the normal structure of the hippocampus. (C & D) VIT C + GSH treated rat brain and hippocampus with an almost similar structure as the controls. (E & F) Lead-exposed rat brain with pyknosis of brain cells (arrow) and areas of necrosis (arrowhead) The hippocampus appears more or less close to the normal. (G & H) VIT C + GSH + Pb treated rat brain and hippocampus showing pyknosis of brain cells (arrow) and less marked areas of necrosis (arrowhead). The hippocampus appears more or less close to the normal (H&E: X 200). After 2 weeks; (A) Control rat brain section shows the normal nerve cells with dispersed chromatin and basophilic cytoplasm. (B) Control rat brain section showing the normal structure of the hippocampus. (C & D) VIT C + GSH treated rat brain and hippocampus with an almost similar structure as the controls. (E & F) Lead-exposed rat brain with widespread pyknosis of brain cells (arrow) and areas of necrosis (arrowhead). The hippocampus appears more or less close to the normal and (G & H) VIT C + GSH + Pb treated rat brain showing pyknosis of brain cells (arrow) and areas of necrosis (arrowhead). The hippocampus: appears more or less close to the normal (H&E: X 200). After 4 weeks; (A) Control rat brain section shows the normal nerve cells with dispersed chromatin and basophilic cytoplasm. (B) Control rat brain section showing the normal structure of the hippocampus. (C & D) VIT C + GSH treated rat brain and hippocampus with an almost similar structure as the controls. (E & F) Lead-exposed rat brain with widespread pyknosis of brain cells (arrow) and areas of necrosis (arrowhead). The hippocampus shows widespread nuclei pyknosis (tailed arrow). (G & H) VIT C + GSH + Pb treated rat brain showing pyknosis of brain cells (arrow) and areas of necrosis (arrowhead). The hippocampus shows widespread nuclei pyknosis (tailed arrow.)(H&E: X 200). After 6 weeks; (A) Control rat brain section shows the normal nerve cells with dispersed chromatin and basophilic cytoplasm. (B) Control rat brain section showing the normal structure of the hippocampus. (C & D) VIT C + GSH treated rat brain and hippocampus with an almost similar structure as the controls. (E & F) Lead-exposed rat brain with widespread pyknosis of brain cells (arrow) and areas of necrosis (arrowhead). The hippocampus shows widespread nuclei pyknosis (tailed arrow). (G & H) VIT C + GSH + Pb treated rat brain showing pyknosis of brain cells (arrow) and areas of necrosis (arrowhead). The hippocampus shows widespread nuclei pyknosis (tailed arrow.)(H&E: X 200).

Brain sections for the cerebrum and hippocampus showed normal nerve cells with dispersed chromatin and basophilic cytoplasm in the control and vitamin C + glutathione-supplemented groups. Lead acetate toxicity induced pyknosis and areas of necrosis in brain cells at the first week of treatment. By the fourth week of lead exposure, pyknosis and necrosis are widespread in brain cells, accompanied by a widespread nuclei pyknosis in the hippocampus, which is more pronounced after 6 weeks of treatment (Fig. 8). In contrast, fortification with vitamin C and glutathione reduces these influences to some extent.

## 4 Discussion

Lead acetate is a widespread toxin. It is a primary global health concern, particularly in areas with heavy industrial pollution and environmental exposure, causing severe and often irreversible organ damage [41]. It triggered a marked decrease in the body weight of rats (Fig. 1A). Bodyweight loss is an essential indicator of the disruption in a rat's general health, and organ weight degradation is vital in determining organ toxicity [42]. The effect of lead acetate is dose-dependent [43]. Another study [44] found that lead acetate exposure at a dose of 1.0 mmol as the sole drinking fluid for 4 weeks did not affect the weight gain of female Wistar rats. Accordingly, they concluded that undernutrition can be ruled out as a cause of the observed changes. Lead binds to appetite-depressant receptors in the gastrointestinal tract, causing decreased food consumption and poor growth [45].

Lead-induced significant elevations in rat liver, kidney, brain, and spleen weights, accompanied by testicular weight reduction. Our results were in line with those reported earlier [46]. The observed increase in the relative organ weight was believed to be caused by cell hyperplasia, apoptosis, and necrosis, which could be linked to the buildup of lipids in the four organs [47]. The result in Fig. 4 emphasizes lead acetate's apoptotic and necrotic action on hepatocytes. It was indicated that chronic lead exposure induced hepatocyte proliferation inflammatory and cholestatic responses [48], which resulted in liver enlargement. Bedossa et al. [49] suggested that lead-induced type I collagen accumulation in hepatic stellate cells promoted hepatic fibrosis impaired by vitamin C supplementation [50].

Rat kidney cells showed a notable accumulation of lipids following lead intoxication [51]. Additionally, they noted that the kidney's dry weight increased with body weight, which a nutritional disruption may have brought on. An extensive examination of the carcinogenic potential of lead salts overall showed that lead acetate causes cancer in rats or mice, with the kidney being the most significant and potentially the organ of interest [52].

Lead inhibited the production of IFNγ in splenic CD4^+^ T cells. It inhibited the IFNγ-dependent Jak1/STAT1 signaling in LTo cells and endothelial cells within the spleen, thereby resulting in enhanced NF-κB signaling, increased proliferation of LTo and endothelial cells, and ultimately leading to splenomegaly [53]. Chronic lead exposure can result in morphological disorders (such as decreased seminiferous tubular diameter, peritubular fibrosis, testicular weight, seminal vesicle loss, and apoptotic-related decrease in germ cell population, as well as functional disorders such as decreased testosterone synthesis [54].

The findings revealed that lead acetate significantly reduced plasma activities of superoxide dismutase and catalase, accompanied by significant (p < 0.05) elevation in plasma malondialdehyde and nitric oxide levels. These observations aligned with those Ilesanmi et al. reported [3]. The primary mechanism underlying lead acetate-induced oxidative stress is the increased generation of reactive oxygen species (ROS), resulting from the disruption of mitochondrial respiratory chain enzymes. The ROS produced reacts with biomolecules to produce peroxide products like protein carbonyl and lipid peroxide, exacerbating the burden on the oxidative defense system as seen by the low levels of SOD and CAT (Fig. 2A and B). Heavy metal-mediated oxidative damage may be linked to the expression of active nitric oxide in red blood cells, contributing to nitrite homeostasis [55]. The elevation of nitric oxide can alter the deformation capacity of cells in response to heavy metal exposure [56].

One of the most critical indicators of oxidative stress is malondialdehyde (MDA), produced when free radicals break down membrane lipids [57]. It was shown that elevated plasma MDA could be utilized as an indicator for free radicals' degradation of liver parenchyma [58]. This explains the significant elevation in plasma GOT and GPT enzymes under lead toxicity due to the enzyme leakage from inflamed and necrotic hepatocytes into the bloodstream, inducing liver injury [59]. It was reported that increased plasma creatinine concentration during hepatic injury suggested that renal perfusion was altered as a result of splanchnic vasodilation linked to portal hypertension, leading to hepatorenal dysfunction [60]. Cardenas and Gines [61] showed that the combined significant elevations in plasma GPT, GOT, and creatinine levels might be linked with hepatic failure. It is noteworthy to note that lead causes both functional and structural hepatorenal disorders (Table 1 and Fig. 5, Fig. 6).

Lead acetate-induced cytoplasmic vacuolation, nuclear pyknosis, engorgement of the central veins, fatty degeneration, disruption of normal hepatocyte structure (Fig. 5), and apoptotic DNA fragmentation in liver cells (Fig. 3A and B). It also induces hepatic early and late apoptosis and necrosis (Fig. 4). It has been demonstrated that cellular vacuolation serves as a defense mechanism against harmful substances [62]. Signs of liver damage after lead treatment include lymphocytic infiltration and sinusoidal congestion [63]. It has been previously reported that poor venous outflow causes blood to stagnate in the capillaries, which leads to congestion [64]. The observed necrosis and pyknosis in the present investigation may be due to ischemia [65], characterized by a decrease in blood flow that impairs the availability of oxygen and nutrients to the liver tissue under lead toxicity. Amorphous eosinophilic cytoplasm may be the first indication of hepatocyte necrosis in the sequence of shrinkage and nuclei dissolving, which may be followed by apoptotic alteration, organelle swelling, particularly the mitochondria and endoplasmic reticulum, and rupture of lysosomes [66]. The observed necrosis of the hepatocytes brought on by long-term exposure to lead may be a sign of oxidative stress caused by glutathione depletion in these cells. As observed in the results of the present study, the cytoplasmic swelling with hydropic degeneration might be associated with the release of lysosomal hydrolytic enzymes, leading to cytoplasmic degeneration and macromolecular accumulation [67].

Results (Fig. 6) indicated that lead-induced glomerulus atrophy, cytoplasmic vacuolation, and obliterated lumens of proximal renal tubules lead to inflammation, fibrosis, and the obliteration of tubular lumens. Glomerulus atrophy compromises the kidney's ability to effectively filter blood, leading to impaired renal function and potentially contributing to the development of renal failure [68]. Cytoplasmic vacuolation can affect the normal cellular processes of the kidney, potentially disrupting cellular metabolism and contributing to overall renal dysfunction [69]. The obliteration of tubular lumens disrupts the normal flow of filtrate within the kidney, impairing reabsorption processes and contributing to renal dysfunction [70]. Mild parenchymal cell degeneration was reported in rats' kidneys after exposure to lead acetate [71]. Lead acetate resulted in tubular wall thinning and indications of tubular cell lining degeneration [72]. Lead exposure was shown to have nephrotoxic effects on renal cortical tissue based on changes in proximal tubular cells [73].

Testicular tissue is affected by lead acetate exposure (Fig. 7), where the germinal epithelium is affected by intraepithelial vacuolation, lacking the spermatogenic series in the tubular lumen. Rats exposed to lead acetate showed comparable alterations and an aggregation of immature cells in the tubular lumen [74]. Mice exposed to lead acetate showed more pronounced degenerative alterations in their testicular tissues, as well as an increase in sperm head abnormalities [75]. It was shown that exposure to lead promoted fibrotic changes in testicular structure, including a noticeable deposition of collagen fibers [76]. Lead exposure induced fibrous thickening of the seminiferous tubular basement membrane, interstitial tissue of the epididymis, and tunica albugenia [77]. Lead stimulates macrophages, which initiate fibrosis [78].

The results revealed a significant decrease in sperm count and sperm motility percentage, especially after 6 weeks of exposure, accompanied by a substantial elevation in the rate of abnormal sperm morphology (Table 2). Lead exposure can impact libido and semen quality by reducing sperm count, motility, viability, and integrity, while also promoting sperm morphological abnormalities [79]. Leon and Pacheco [80] showed that lead directly affects the quality of semen, accompanied by elevated plasma lead levels, which was in line with the data reported in Fig. 3C, where lead exposure-induced elevations in lead levels in whole blood, liver, kidney, and urine. These data suggest that the body adapted to lead poisoning by eliminating lead in the urine and storing the excess in different body organs.

Lead exposure resulted in the development of necrotic patches and widespread pyknosis among brain cells. These changes may be linked to neural oxidative stress, mitochondrial dysfunction, inflammatory responses, and calcium dysregulation enhanced by lead exposure [81]. Notably, extensive nuclei pyknosis manifested prominently in the hippocampus, as illustrated in Fig. 8. Seddik et al. [82] reported an elevation in Pb levels, particularly in the hippocampus, compared to the amygdala and serum. These experimental observations prove that Pb can traverse the blood-brain barrier, primarily impacting the hippocampus. The hippocampus and the amygdala play crucial roles in emotional processing, learning, and memory, so they emerge as potential target structures for the neurotoxic effects induced by Pb poisoning [83]. Results indicated an elevation in brain weight under lead exposure (Fig. 1D). Lead exposure can cause brain inflammation, resulting in cerebral edema and gliosis [84]. The swelling of the brain caused by excess fluid accumulation can lead to an increase in brain weight. The inflammatory response to lead can disrupt the blood-brain barrier by mobilizing calcium and activating protein kinases in endothelial cells, causing fluid leakage into brain tissue [85]. Lead exposure is known to cause chronic neuroinflammation, characterized by the stimulation of microglia and astrocytes and infiltration of peripheral immune cells into the brain [86].

The data suggested that the administration of vitamin C and glutathione alleviates the toxic effects of lead acetate. As a water-soluble vitamin, vitamin C plays a crucial role in the proper functioning and restoration of various tissue types, acting as an endogenous antioxidant and a cofactor in enzymatic reactions [87]. Vitamin C functions as a broad-spectrum free radical scavenger, effectively neutralizing peroxyl and hydroxyl radicals, superoxide anions, singlet oxygen, and peroxynitrite [88]. Given the brain's high energy consumption, elevated metabolic activity, and significant polyunsaturated fatty acid content, vitamin C is crucial for preserving brain tissue. Vitamin C's antioxidant-free scavenging activity protects membranes and their hydrophobic compartment [89]. Accordingly, it has potent antioxidant defense characteristics [90].

Vitamin C can chelate lead ions, reducing their absorption from the gastrointestinal tract and elevating renal excretion [91]. It resists metabolic breakdown, can reach metal storage sites, promotes metal excretion, and reduces toxicity [92]. Vitamin C reverses oxidative damage caused by lead exposure [7]. It can delay the production of free radicals, disrupting the radical chain reaction, chelating Pb2+ ions, halting further free radical production, and stimulating other endogenous antioxidant defenses [93].

Yousef et al. [94] showed that vitamin C has the potential to mitigate lead acetate-induced hepatorenal toxicity and alleviate the majority of the histopathological alterations associated with lead exposure. Vitamin C helps neutralize reactive oxygen species (ROS) by reducing oxidative stress and minimizing associated complications [19]. Vitamin C mitigates the reactivity of free radicals by donating electrons, thereby neutralizing their harmful effects [95]. It serves as a hepatocellular free radical scavenger and prevents hepatic glutathione depletion in rats [96].

According to Fraga et al. [97], vitamin C supplementation for rats led to increased dendritic spine density by stimulating the Akt pathway and its downstream enzyme 70S6K. This enzyme phosphorylates and modulates the activity of ribosomal protein S6, a critical component of the molecular translation system. Furthermore, it has been demonstrated that the promotion of Akt and phosphoinositide 3-kinase (PI3K), followed by the subsequent activation of 70S6K mediate the antidepressant-like impacts stimulated by the oral administration of vitamin C. This mechanism occurs concurrently with a marked increase in postsynaptic density protein 95 (PSD-95) levels within the hippocampus of young adult mice [98]. Vitamin C can reduce blood lead levels with increased urinary lead excretion [99], restore biochemical indices, and normalize the histological architecture of the liver, kidney, testis, and brain tissues (Fig. 5, Fig. 6, Fig. 7, Fig. 8).

Concerning glutathione, Mirkovic et al. [100] showed that reduced glutathione (GSH) is a desirable supplement for helping rats exposed to lead poisoning to detoxify reactive oxygen species. On the other hand, GSH binds lead (Pb) weakly [101], and its ability to reduce Pb toxicity is limited [102], which can be explained by GSH's low affinity for Pb [103]. Glutathione has amplified the cells' capacity to sequester toxins and avert oxidative cellular damage [104]. It has been suggested that GSH can chelate and detoxify metals as soon as they enter the cell [105]. Eliminating hazardous oxygen species is another way that GSH contributes to metal detoxification. Moreover, glutathione serves as a substrate for GSH peroxidases, which are enzymes that can repair peroxidative damaged membranes and extract hydrogen peroxide from cells [106].

GSH maintains the stability of the plasma membrane under lead stress [107]. Khan et al. [108] showed that GSH normalizes excessive ROS levels, optimizes the activities of antioxidant enzymes, and decreases Pb uptake. Perez et al. [109] reported that Pb exposure reduced intracellular GSH levels, indicating that intracellular GSH plays a role in Pb detoxification. GSH protects hepatocytes, erythrocytes, and other cells against lead toxicity [110]. The central nervous system contains binding sites for GSH. Thus, GSH is considered a neuromodulator [111].

The synergistic action of vitamin C and glutathione against lead acetate toxicity may involve several mechanisms. The potential mechanism is the renewal of an antioxidant that has oxidized by another, which enhances the overall antioxidant capacity of the body [112]. Additionally, variations in antioxidant partitioning across different biological systems may make the synergistic effect of these compounds more pronounced and beneficial for various organs [113]. Combining free radical scavenging and lead chelation activities provides two distinct protective routes, contributing to their effectiveness [114]. Furthermore, after oxidation, vitamin C and glutathione may enhance the production of additional antioxidant compounds, including phenolics, dimers, or adducts, which can further reduce lipid peroxidation induced by lead acetate [115]. The co-supplementation of these compounds also improves the detoxification process, where glutathione assists in detoxifying mercury, while vitamin C supports this process by reducing oxidative stress [116]. This combined antioxidant action helps to reduce cellular damage caused by oxidative stress and mercury toxicity, thereby safeguarding essential cellular constituents such as lipids, proteins, and DNA [117].

Moreover, vitamin C and glutathione may influence protein modification within cells, enhancing their function in response to oxidative stress [114,118]. Additionally, glutathione supports autophagy [119], a process that removes damaged cells and toxins, thereby minimizing the detrimental effects of lead exposure. Vitamin C may also improve ion transport across cell membranes [120], contributing to cellular stability during lead exposure. Activating cellular signaling pathways (e.g., the AKT pathway) by vitamin C could further enhance cell survival, equipping cells to cope better with lead-induced stress [121,122]. Vitamin C can promote endogenous glutathione production [123,124], bolstering the antioxidant defenses against lead toxicity.

Vitamin C and glutathione co-supplementation show several advantages over conventional chelating agents for lead protection. Cost-effectiveness is achieved using lower-cost raw materials and simpler synthesis processes, reducing production expenses compared to EDTA and CaNa₂ EDTA [125,126]. Vitamin C and glutathione supplements may offer protective effects against lead toxicity, which could help reduce the frequency of use of traditional chelating agents [127,128]. The favorable safety profile of vitamin C and glutathione supplements shows fewer adverse effects, a key advantage over chelators such as penicillamine and succimer, EDTA, which are associated with allergic and hematologic reactions [[128], [129], [130]]. Physiological and biochemical assessments (body and organ weights, SOD, CAT, MDA, NO, plasma lead, apoptotic DNA fragmentation, annexin V, caspase-3, and fibrosis) confirm that combined vitamin C and glutathione supplementation effectively reduces plasma lead levels while protecting organs like the liver, kidneys, testes, and brain, as supported by histopathological analysis (Fig. 5, Fig. 6, Fig. 7, Fig. 8). Lastly, Vitamin C and glutathione could be vital in alleviating lead toxicity in various environmental matrices, suggesting potential benefits that merit investigation alongside conventional chelators [[131], [132], [133]]. While commercial chelators are essential for life-saving interventions in acute lead poisoning cases [128], using antioxidants as preventive measures may be more effective in reducing the risks associated with chronic lead exposure [25].

While vitamin C and glutathione provide sustained antioxidant protection against lead toxicity over the study period, their limited reusability suggests that developing slow-release formulations could enhance their durability and effectiveness in long-term, chronic exposure scenarios.

Our study uniquely examines the combined antioxidant effects of vitamin C and glutathione, potentially offering synergistic benefits. This co-administration approach enhances protective effects, providing a more effective strategy for reducing oxidative damage and preserving organ function in lead-exposed animals.

## 5 Conclusion

In conclusion, this study indicates that lead exposure induces significant physiological and histopathological disturbances, including impaired organ function, altered biochemical indices, and increased tissue lead accumulation. Our findings reveal that co-supplementation with vitamin C and glutathione provides a protective effect by mitigating many of these adverse effects. Specifically, this combination effectively improved key biochemical markers, reduced lead accumulation, and preserved vital organs' structural and functional integrity.

These results support the hypothesis that vitamin C and glutathione offer a synergistic defense against lead toxicity, potentially enhancing overall antioxidant capacity and improving heavy metal detoxification. This work suggests an approach for lead toxicity mitigation, highlighting the combined use of antioxidants as a possible alternative or adjunct to conventional chelation materials.

Future studies should explore optimal dosing strategies, evaluate the efficacy of long-term and fractionated doses, and conduct comparative studies between vitamin C and glutathione and conventional chelators to identify the most effective options for prevention and treatment across various lead toxicity scenarios. Additional investigations could also elucidate the mechanisms underlying the observed protective effects, positioning vitamin C and glutathione as viable alternatives or complements in managing lead toxicity.

## Ethics statements

The experimental protocol was reviewed and approved by the Research Ethics Committee of the Faculty of Science at Arish University with approval number “ARU/SF.02". Our experimental procedures comply with the guidelines outlined in the 8th edition of the guide for the care and use of laboratory animals with strict adherence to ethical guidelines. The study complies with all regulations.

## Data availability statement

Data will be made available on request.

## Funding

This research received no specific grant from the public, commercial, or not-for-profit funding agencies.

## Declaration of competing interest

The authors declare that they have no known competing financial interests or personal relationships that could have appeared to influence the work reported in this paper.

## List of abbreviations

ALT: Alanine aminotransferase
AST: Aspartate aminotransferase
CAT: Catalase
DNA: Deoxyribonucleic acid
EDTA: Ethylenediaminetetraacetic acid
GOT: Glutamic oxaloacetic transaminase
GPT: Glutamic pyruvic transaminase
GPX: Glutathione peroxidase
GSH: Reduced glutathione
GSR: Glutathione disulfide reductase
GSSG: Glutathione disulfide
GSTs: Glutathione-S-transferases
MDA: Malondialdehyde
Pb: Lead
RNS: Reactive nitrogen species
ROS: Reactive oxygen species
SD: Standard deviation
SOD: Superoxide dismutase
Vit C: Vitamin C
